# Patient-reported outcomes following surgery for adolescent idiopathic scoliosis performed in adolescence versus adulthood

**DOI:** 10.1308/rcsann.2024.0067

**Published:** 2024-09-03

**Authors:** A Lloyd, I Harding, A Cole, A Gardner

**Affiliations:** ^1^ The Royal Orthopaedic Hospital NHS Foundation Trust, UK; ^2^ North Bristol NHS Trust, UK; ^3^ Sheffield Children’s NHS Foundation Trust, UK

**Keywords:** AIS, ASA, Outcomes, SRS-22r, HRQoL

## Abstract

**Introduction:**

The Scoliosis Research Society 22r (SRS-22r) questionnaire is a proven tool in assessing healthcare-related quality of life (HRQoL) in idiopathic scoliosis and is the adopted patient-reported outcome measure for the deformity pathway recorded into the British Spinal Registry (BSR). Surgery for adolescent idiopathic scoliosis (AIS) is performed frequently in teenagers; however, patients also present with curves in the surgical range into adulthood. This work aimed to assess HRQoL differences between patients following surgery for AIS performed in adolescence and adulthood using SRS-22r data collected from the BSR.

**Methods:**

An anonymised BSR search of pre- and postoperative SRS-22r scores for patients with diagnoses of AIS and adult idiopathic scoliosis was conducted. Data from all subdomains were compared preoperatively and at the two-year postoperative timepoint.

**Results:**

Preoperative SRS-22r scores were analysed for 1,912 patients with AIS and 65 with adult idiopathic scoliosis. Patients with adult idiopathic scoliosis had significantly lower preoperative SRS-22r scores in all subdomains (*p*<0.05). By two years postoperatively, both groups of patients had improved SRS-22r scores significantly compared with baseline in all subdomains (*p*<0.001). A cross-group analysis revealed patients with AIS had significantly better function scores years postoperatively than their adult counterparts (*p*=0.005).

**Conclusions:**

This work confirms there are benefits following surgery for AIS in improving HRQoL, but has also provided HRQoL data in adult patients, who again show similar improvements following surgery from baseline. This is of value when counselling patients regarding anticipated benefits of surgery performed in childhood and adulthood.

## Introduction

A key element of success of intervention in those with spinal deformity is the affected individuals' impression of their condition.^1^ The personal perception of health-related quality of life (HRQoL) can also then present challenges in quantifying and evaluating both disease burden and efficacy of treatment.^1^ Since its original description by Haher^2^ through to its latest iteration, modified by Asher and colleagues,^3^ the Scoliosis Research Society 22 revised (SRS-22r) questionnaire has become a proven and reliable tool in assessing HRQoL in idiopathic scoliosis in both adolescents and adults.^4^ The SRS-22r is sensitive to changes following surgical intervention^5^ and has been reported as the patient-reported outcome measure used most in studies of patients with scoliosis.^6^

Adolescent idiopathic scoliosis (AIS) accounts for the vast majority of cases of scoliosis in the skeletally immature, with a prevalence in the general population of between 0.5 and 5.2%.^7^ Consideration of surgical intervention in the treatment of AIS is dependent on a number of factors, with particular weight given to the potential for progression of the spinal deformity.^8^ Risk of progression in AIS is particularly marked in the skeletally immature, with general consensus indicating surgery could be considered once a curve reaches 45–50°.^9^ However, the risk of progression and the potential for poor clinical outcome is not obviated with the attainment of skeletal maturity, with a potential for curve progression existing into adulthood if a curve approaches 50° at the end of skeletal growth.^8^

Once a decision to proceed with surgery for a progressive curve has been agreed among all parties concerned, a further dilemma for the operating surgeon is the optimal time for intervention. Proponents of early surgery consider that intervening before the onset of clinical sequelae of untreated progressive AIS,^10^ in addition to technically favourable elements to the surgery such as reduced requirement for osteotomies and reduced estimated blood loss,^11^ as justifiable reasons for surgery. However, there is lack of consensus in the surgical community, with opponents arguing that the rates of progression, iatrogenic cessation of spinal growth caused by a spinal fusion, and psychosocial morbidity, including the impact on education, as legitimate reasons to delay surgery into adulthood.^12,13^

It is important to recognise that scoliosis in adulthood occurring as a result of untreated AIS (so called ‘adolescent scoliosis of the adult’ (ASA)^14^), represents a distinct and different clinical entity from de novo degenerative scoliosis. Whereas de novo degenerative scoliosis is typically characterised by pain and disability, the natural history of ASA is less certain, with conflicting reports particularly regarding rates of back pain in comparison with the general population.^14^ Recent works researching idiopathic scoliosis have reported that delaying surgery into adulthood is associated with curve progression leading to increased fusion levels and decreased curve correction,^12^ in addition to increased major complication rates^15^ in comparison with surgical intervention in adolescence. However, little is known regarding the results of early versus delayed surgery for AIS on patient-reported outcomes and HRQoL.

The aim of the present study was to evaluate both preoperative and postoperative HRQoL using the SRS-22r measurement tool in patients who have undergone surgery for idiopathic scoliosis in adolescence (<18 years of age) and adulthood (≥18 years of age). This work would therefore add further evidence to answering the question of whether surgical intervention influences subsequent HRQoL in patients with these forms of idiopathic scoliosis in addition to making an inference as to whether timing of surgery performed either in adolescence or adulthood has any influence on subsequent HRQoL.

## Methods

This was a retrospective analysis of prospectively collected, multicentre data entered onto the British Spinal Registry (BSR). The BSR is an electronic database of clinical and patient-reported outcome data established in 2012 for patients undergoing operative spinal procedures in the UK. With the permission of the data processor of the BSR from the British Association of Spinal Surgeons (BASS) and British Scoliosis Society (BSS), an anonymised search of preoperative and postoperative SRS-22r outcome data for patients with recorded diagnoses of AIS and ASA was conducted and analysed in May 2020. Postoperative SRS-22r scores were collected at the six-week, six-month, one-year and two-year postoperative timepoints.

The data were analysed as median, range and interquartile range (IQR), lower and upper quartiles. Both intra- and intergroup analyses were performed, with data from all subdomains (function/activity, pain, mental health, self-image and subtotal domains) analysed preoperatively and at the two-year postoperative timepoint using the Wilcoxon rank sum test. Differences in scores between preoperative and two-year postoperative timepoints for each group were determined using the Kruskall–Wallis test and Mann–Whitney *U* test where appropriate.

Alpha for all analyses was set at 0.05. All data analyses were performed using R statistical software version 3.6.1.^16^

## Results

In total, preoperative SRS-22r scores were available for 1912 patients with AIS and 65 patients with ASA. There was a loss to follow-up in both groups at each subsequent postoperative timepoint as shown in Table 1, with both preoperative and postoperative SRS-22r scores available for 865 patients (45.2%) with AIS and 41 patients (63.1%) with ASA at the two-year postoperative timepoint.

**Table 1:** The number of individual SRS-22r scores available for analysis at the preoperative and postoperative timepoints

Timepoint	AIS (*n*; % from baseline)	ASA (*n*; % from baseline)
Preoperative	1,912 (100)	65 (100)
Six-week postoperative	1,367 (71.5)	56 (86.2)
Six-month postoperative	1,290 (67.5)	60 (92.3)
One-year postoperative	1,215 (63.5)	55 (84.6)
Two-year postoperative	865 (45.2)	41 (63.1)

AIS = adolescent idiopathic scoliosis; ASA = adolescent scoliosis of the adult.

Significant differences were observed in the preoperative SRS-22r scores between groups, with lower (worse) scores observed in the ASA cohort in the function, mental health, self-image, pain and overall subtotal domains, as exhibited in Table 2.

**Table 2:** Baseline median preoperative SRS-22r subdomain scores of AIS (*n*=1,912) and ASA (*n*=65) groups

SRS-22r subdomain	AIS scores (IQR as Q1 and Q3,range as minimum to maximum)	ASA scores (IQR as Q1 and Q3,range as minimum to maximum)	*p* value*
Subtotal	3.5 (3.1 to 4.0, 1.6 to 5.0)	3.2 (2.7 to 3.6, 1.7 to 4.5)	<0.0001
Function	4.0 (3.6 to 4.4, 1.3 to 5.0)	3.6 (3.2 to 4.0, 1.4 to 5.0)	<0.0001
Pain	3.6 (3.0 to 4.2, 1.0 to 5.0)	3.0 (2.6 to 3.8, 1.0 to 5.0)	<0.0001
Mental health	3.8 (3.0 to 4.2, 1.0 to 5.0)	3.4 (3.0 to 4.0, 1.8 to 5.0)	0.0337
Self image	3.0 (2.4 to 3.4, 1.0 to 5.0)	2.8 (2.0 to 3.2, 1.0 to 4.0)	0.0095

AIS = adolescent idiopathic scoliosis; ASA = adolescent scoliosis of the adult; IQR = interquartile range.

*Wilcoxon rank sum test.

**Figure 1:**
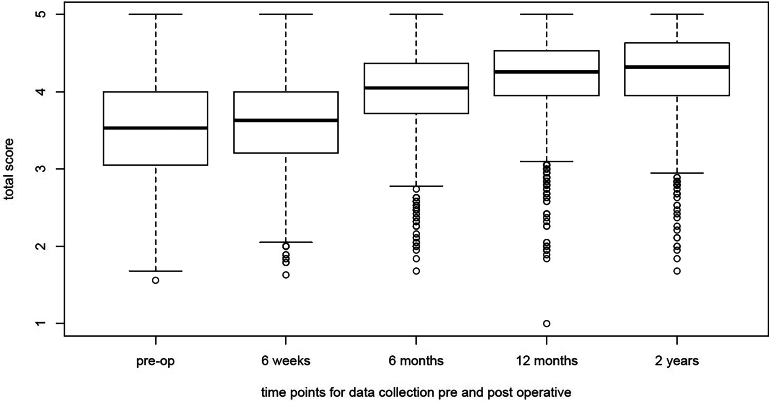
Box and whisker plot of the subtotal SRS-22r scores over two-year total follow-up AIS group. The thick line on the box is the median value. The box represents the IQR and the whiskers are 1.5 times the IQR. Any datapoints outside that are represented as open circles. AIS = adolescent idiopathic scoliosis; IQR = interquartile range.

**Figure 2:**
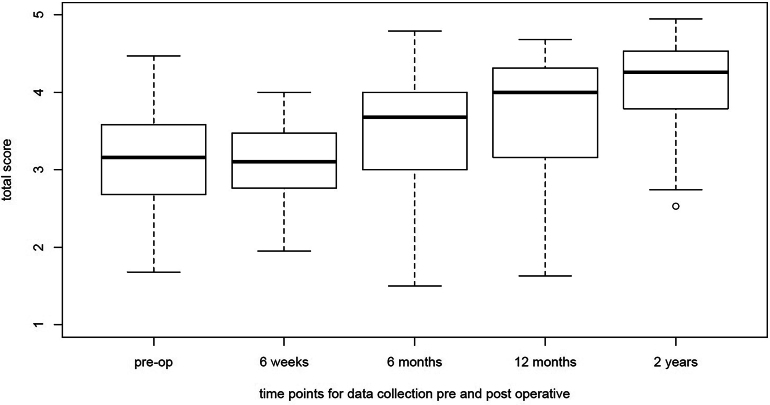
Box and whisker plot of the subtotal SRS-22r scores over two-year total follow-up ASA group. The thick line on the box is the median value. The box represents the IQR and the whiskers are 1.5 times the IQR. Any datapoints outside that are represented as open circles. ASA = adolescent scoliosis of the adult; IQR = interquartile range.

Compared with their respective preoperative scores, patients with AIS displayed improvements in each subdomain at the two-year postoperative timepoint with subtotal scores displayed in Figure 1 (*p*<0.0001 all subdomains). Similarly, patients with ASA also demonstrated improvements in each subdomain at two years postoperatively, with subtotal scores shown in Figure 2 (*p*<0.001 all subdomains).

Analyses of scores between groups at two years postoperatively are displayed in Table 3. No significant differences in the pain, mental health, self image subdomains and overall subtotal SRS-22r scores were observed between groups; however, function scores were significantly higher in the AIS group.

**Table 3:** Median two-year postoperative SRS-22r subdomain scores of AIS (*n*=865) and ASA (*n*=41) groups

SRS-22r subdomain	AIS scores (IQR as Q1 and Q3,range as minimum to maximum)	ASA scores (IQR as Q1 and Q3,range as minimum to maximum)	*p* value*
Subtotal	4.3 (4.0 to 4.6, 1.7 to 5.0)	4.3 (3.8 to 4.5, 2.5 to 5.0)	0.23
Function	4.4 (4.0 to 4.6, 1.6 to 5.0)	4.2 (3.8 to 4.6, 2.0 to 4.8)	0.005
Pain	4.4 (4.0 to 4.8, 1.0 to 5.0)	4.4 (3.8 to 4.8, 2.6 to 5.0)	0.46
Mental health	4.2 (3.6 to 4.6, 1.0 to 5.0)	4.2 (3.6 to 4.6, 2.4 to 5.0)	0.74
Self image	4.4 (4.0 to 4.8, 1.2 to 5.0)	4.2 (3.6 to 4.6, 2.0 to 5.0)	0.22

AIS = adolescent idiopathic scoliosis; ASA = adolescent scoliosis of the adult; IQR = interquartile range.

*Mann-Whitney *U* test.

## Discussion

Despite general consensus of the indications around surgical intervention in patients with AIS,^8,9^ the timing of surgery remains a topic of constant debate in the spinal surgical community. Logical and legitimate arguments for both early and delayed intervention for curves in the surgical range may be found in the literature.^10–13^ Previous research in this area has focussed largely on the technical aspects of early versus delayed surgery in patients with AIS. Yang and colleagues^17^ have reported changes in curve pattern and increased fusion levels when delaying surgery for AIS to adulthood.

More recently, Lonner *et al* have published data demonstrating increased operative time, estimated blood loss and length of hospital stay comparing operative outcomes in patients with AIS with a matched cohort with adult idiopathic curves.^15^ However, a recent systematic review and meta-analysis from Chen *et al*, comparing operative outcomes between patients with AIS and ASA (alternatively termed adult idiopathic scoliosis in their work), concludes significantly improved correction of the major structural curve is seen in patients with AIS versus ASA (68.4% vs 61.4%) but no significant differences were observed in other perioperative or postoperative variables.^18^

This study reports on registry data HRQoL scores in patients who have undergone surgical intervention for AIS and ASA. The data presented have shown that, in comparison with patients with a diagnosis of AIS, those with ASA have lower SRS-22r scores in all subdomains before surgical intervention. This finding is unsurprising in the context of the current understanding of the natural history of AIS from long-term follow-up studies,^9^ with particular regard to increasing rates of back pain experienced by patients whose curve has continued to progress into adulthood.^14,19^ Following surgical intervention, we found that the median SRS-22r scores in all subdomains improved significantly, independent of whether surgery was performed in adolescence or adulthood. This adds to the growing evidence base that supports the efficacy of surgical intervention in improving HRQoL in those with idiopathic scoliosis irrespective of age at operation.^15^

Interestingly, the registry data showed no significant differences in total SRS-22r or most subdomain scores two years postoperatively when comparing outcomes between groups. The only significant difference identified was in the function subdomain, which found that patients with AIS tended to have higher scores than those with ASA. Although we cannot directly infer from registry data the reasons behind this observed finding, given the greater general activity levels observed in adolescence, the observed difference may simply represent the natural proclivity for activity in youth. Of particular interest, both the two-year median postoperative scores in the intragroup as well as the intergroup analysis exceeded the minimal detectable measurement difference values in each subdomain analysis as previously advocated by Kelly *et al*,^20^ indicating that the observed improvements were both clinically as well as statistically significant.

The strengths and limitations of this study are linked inherently to the dataset from the BSR. The strengths of this study’s use of registry data include analysis of a large, multicentre cohort of patient-reported outcome measures, which form a critical element of evaluating success of surgical intervention^1^ and are of increasing interest to commissioners in evaluating the provision of services in healthcare. The limitations of use of these data are the reliance on the accurate input of data at source and the high attrition rates across the observed follow-up period, which limits the general applicability of the study findings due to possible selection bias.

Unfortunately, the current iteration of the BSR also precludes the possibility of a further substratification by age into more specific groups, which prevents a more detailed analysis and perhaps could be addressed in future modifications to the registry.

It is well accepted that adult de novo degenerative deformity represents a totally separate and incomparable clinical entity from AIS treated in adulthood, and therefore accurate diagnosis entry at source input is also an assumption and potential source of bias. This critical distinction between an evolved adolescent curve treated in adulthood and a degenerative de novo deformity has also been highlighted as a potential source of bias in the systematic review and meta-analysis reported by Chen and colleagues,^18^ summarising the lack of a clear definition and/or age differentiation that limits the current literature focusing on this subject. We would recommend that this be the focus of a future work that would be of benefit to further research in this area.

In conclusion, this study reports that HRQoL, as measured by the SRS-22r tool, improves following surgical intervention in both patients with AIS and adult idiopathic scoliosis. Postoperative SRS-22r scores were not found to differ significantly between patients across both groups, with the exception of the function subdomain, where patients with AIS were significantly better than those in the ASA group. This work provides additional evidence to support decision makers in the timing of surgical intervention for patients with AIS, showing that HRQoL is similar between the two groups at two years after surgery and making the decision for timing of surgery dependent on other factors.

## Data Availability

Data are available on request. Level of evidence: Level 2.
